# Analysis of SteraMist ionized hydrogen peroxide technology in the sterilization of N95 respirators and other PPE

**DOI:** 10.1038/s41598-021-81365-7

**Published:** 2021-01-21

**Authors:** Avilash K. Cramer, Deborah Plana, Helen Yang, Mary M. Carmack, Enze Tian, Michael S. Sinha, David Krikorian, David Turner, Jinhan Mo, Ju Li, Rajiv Gupta, Heather Manning, Florence T. Bourgeois, Sherry H. Yu, Peter K. Sorger, Nicole R. LeBoeuf

**Affiliations:** 1 Greater Boston Pandemic Fabrication Team (PanFab) c/o Harvard-MIT Center for Regulatory Science, Harvard Medical School, Boston, MA USA; 2 Harvard-MIT Division of Health Sciences and Technology, Cambridge, MA USA; 3 Department of Systems Biology, Harvard Medical School, Boston, MA USA; 4 Harvard-MIT Center for Regulatory Science, Harvard Medical School, Boston, MA USA; 5 Computational Health Informatics Program, Boston Children’s Hospital, Boston, MA USA; 6 Department of Building Science, Tsinghua University, Beijing, China; 7 Department of Nuclear Science and Engineering and Department of Materials Science and Engineering, MIT, Cambridge, MA USA; 8 Dana-Farber Cancer Institute, Boston, MA USA; 9 Harvard Medical School, Boston, MA USA; 10 Department of Radiology, Massachusetts General Hospital, Boston, MA USA; 11 Department of Dermatology, Yale University School of Medicine, New Haven, CT USA; 12 Department of Dermatology, Brigham and Women’s Hospital, Boston, MA USA

**Keywords:** Occupational health, Infectious diseases, Viral infection

## Abstract

The COVID-19 pandemic has led to widespread shortages of personal protective equipment (PPE) for healthcare workers, including of N95 masks (filtering facepiece respirators; FFRs). These masks are intended for single use but their sterilization and subsequent reuse has the potential to substantially mitigate shortages. Here we investigate PPE sterilization using ionized hydrogen peroxide (iHP), generated by SteraMist equipment (TOMI; Frederick, MD), in a sealed environment chamber. The efficacy of sterilization by iHP was assessed using bacterial spores in biological indicator assemblies. After one or more iHP treatments, five models of N95 masks from three manufacturers were assessed for retention of function based on their ability to form an airtight seal (measured using a quantitative fit test) and filter aerosolized particles. Filtration testing was performed at a university lab and at a National Institute for Occupational Safety and Health (NIOSH) pre-certification laboratory. The data demonstrate that N95 masks sterilized using SteraMist iHP technology retain filtration efficiency up to ten cycles, the maximum number tested to date. A typical iHP environment chamber with a volume of ~ 80 m^3^ can treat ~ 7000 masks and other items (e.g. other PPE, iPADs), making this an effective approach for a busy medical center.

## Introduction

The COVID-19 pandemic has led to widespread shortages in personal protective equipment (PPE) for clinicians and first responders. Shortages in filtering facepiece respirators (FFRs) such as N95 “masks,” which are certified to filter 95% of airborne particles at 0.3 µm, are particularly problematic because these normally single-use items are a mainstay of infection control. It has been widely reported that the US Department of Health and Human Services (HHS) anticipates a need for as many as 3.5 × 10^9^ N95 masks^1^ in 2020–2021 for US use alone, but estimates of total available supply are far short of that number^2^. The consequent need for N95 mask sterilization and subsequent reuse is therefore likely to continue for the foreseeable future. The possibility that disposable N95 masks could be sterilized and reused was raised 15 years ago as a strategy to address shortages arising from medical emergencies^3–5^, but following an FDA-funded study by the Battelle Memorial Institute^6^, relatively little subsequent research has been performed on the topic^7–9^. Recently, in response to acute N95 mask shortages, multiple strategies for mask sterilization have been proposed and studied, including exposure to ultraviolet (UV) germicidal irradiation, vaporized hydrogen peroxide, moist heat, ethylene oxide, and gamma irradiation^7–13^. In this study, we evaluate a recently developed technology, ionized hydrogen peroxide (iHP), as a method for sterilizing N95 masks and other PPE.

Hydrogen peroxide (H_2_O_2_) is a powerful sterilizing agent that can be used on porous and other surfaces following vaporization or ionization to create a mist containing hydroxyl radicals. Such vaporized or ionized hydrogen peroxide methods (VHP/iHP) are widely used for environmental sterilization across multiple industries including food preparation, healthcare, and life sciences^14–19^. VHP/iHP methods can be used on a wider range of sensitive materials than high temperature methods such as autoclaving. Ethylene oxide (EtO) is another widely used cold sterilization method but use of EtO with face masks has been hindered by concerns about the carcinogenicity and toxicity of residual EtO. In contrast iHP is considered to be safer, as it breaks down to into water. Nonetheless, it is routine to test for the presence of residual H_2_O_2_ post sterilization using an instrument such as a PortaSens II Portable Gas Leak Detector (Analytical Technology, Inc., Collegeville, PA)^20^.

Four distinct VHP/iHP-based H_2_O_2_ sterilization technologies have been commercialized to date and are shown in Table 1. Each technology involves a different approach to generating and delivering the sterilant. In all cases microbial killing is achieved through the reaction of hydroxyl radicals with proteins, nucleic acids and other biomolecules in pathogens. Three VHP-based systems have received emergency use authorization (EUA) from the Food and Drug Administration (FDA) for N95 mask decontamination^21^, even though relatively limited peer-reviewed data is available^11^, particularly from non-commercial third parties. As a consequence, it is difficult for infection control teams in hospitals and other healthcare providers to evaluate and compare these systems. The absence of data on the post-sterilization performance of different makes and models of N95 masks is also limiting. The Brigham and Women’s Hospital (BWH; Boston MA) Incident Command, which is involved in this study, currently has on hand over 30 models of N95 masks from three manufacturers.

**Table 1:** Comparison of commercial sterilization technologies that use vaporized and ionized hydrogen peroxide.

Company	Products	Technology	Technology of delivery	Existing Use for N95 Sterilization
Bioquell	Bioquell Clarus C; Bioquell Z-2; Bioquell ProteQ	**HPV** *Hydrogen Peroxide Vapor*	30–35% liquid H_2_O_2_ vaporized and delivered into a chamber; saturated H_2_O_2_ condenses on surfaces^6,14,15,18^	EUA granted (3/28/2020) to Batelle to use Bioquell as part of its Critical Care Decontamination System for up to 20 cycles of N95 mask reuse^39^
Steris Corporation	STERIS V-PRO 1 Plus, maX and maX2 Low Temperature Sterilization Systems	**VHP** *Vaporized Hydrogen Peroxide*	30–35% liquid H_2_O_2_ is vaporized and delivered into a dehumidified chamber; concentration is held below condensation point^16^	EUA granted (4/10/2020) to Steris Corporation for STERIS V-PRO 1 Plus, maX and maX2 Low Temperature Sterilization Systems for up to 10 cycles of single-user reuse^40^
Advanced Sterilization Products (ASP)	STERRAD 100S H_2_O_2_ Gas Plasma Sterilizer; STERRAD NX H_2_O_2_ Gas Plasma Sterilizer; STERRAD 100NX H_2_O_2_ Gas Plasma Sterilizer	**HPGP** *Hydrogen Peroxide Gas Plasma*	58–60% liquid H_2_O_2_ is vaporized into a chamber; radiation frequency energy is targeted into the chamber, exciting the H_2_O_2_ to a low-temperature plasma state^7,21^	EUA granted (4/12/2020) to ASP for STERRAD 100S Cycle, STERRAD NX Standard Cycle, or STERRAD 100NX Express Cycle for up to two cycles in Tyvek pouching for single-user reuse^38^
TOMI	SteraMist Binary Ionization Technology (BIT)	**iHP** *Ionized Hydrogen Peroxide*	7.8% aqueous H_2_O_2_ aerosolized. 0.05–3 µm droplets are pushed past a cold plasma field generated by two electrodes and ionized into hydroxyl radicals^41^	The topic of this study. Currently being investigated for sterilization of PPE for re-use in academic medical centers

This study focuses on the use of iHP as a N95 mask sterilization method, specifically the SteraMist Binary Ionization Technology (BIT) from TOMI (Beverly Hills, CA). iHP was registered with the Environmental Protection Agency (EPA) in 2015 for use in health care, life sciences, food safety, and other settings (appearing on EPA lists G, H, K, L, and M). Most recently it was added to EPA List N: Disinfectants for Use Against SARS-CoV-2, for use on hard, nonporous surfaces^22^. The active ingredient in iHP is 7.8% aqueous H_2_O_2_ which is flowed past a plasma arc and dispersed into a treatment chamber as a mist of micron-sized liquid droplets. The iHP method used in this study differs from the VHP method used by Battelle (based on Bioquell technology) in its “Critical Care Decontamination System” for sterilization of N95 masks primarily because iHP uses a ~ 5-fold lower H_2_O_2_ concentration (7.8% vs. 30–35%; Table 1). Use of a lower H_2_O_2_ concentration is possible because flowing H_2_O_2_ through a plasma arc in an iHP system directly generates hydroxyl radicals, which are a powerful oxidizing agent that functions as the active sterilant (Table 1); in vapor-based systems hydroxyl radicals are generated by spontaneous decomposition of H_2_O_2_. iHP is commercially available in two implementations: a handheld sprayer device (“Surface Unit”) and an environmental unit (“Environment System”).

The environment system used in the current research was previously installed at the Dana-Farber Cancer Institute (DFCI; Boston, MA) animal research facility for use in sterilizing incoming equipment and materials; installations of this type are quite common. In this study, following cycles of sterilization, masks were tested for three critical features: (1) sterility, as measured by the inactivation of bacterial spores contained in biological indicators; (2) filtration efficiency, measured both by aerosolized 75 nm NaCl particles and by 0.3–1 µm ambient particulate matter; and (3) fit, using a PortaCount quantitative fit test apparatus. Multiple sterilization cycles were completed to assess mask durability. Sterilization of other PPE items such as face shields and hoods and hoses for Powered-Air Purifying Respirators (PAPRs) was also explored. Testing was performed at the DFCI, MIT and ICS Laboratories (http://www.icslabs.com/; Brunswick, OH) a commercial laboratory accredited to perform testing to NIOSH/ISO/IEC standards^23^.

## Methods

### Selection of N95 respirators and other PPE samples

A total of 83 N95 masks representing five models from three manufacturers (3M 1860, Kimberly-Clark [KC]/Halyard 46767 “duckbill,” Gerson 2130, 3M 8210, and 3M 9210/37021) were selected for testing as a representative sample of the N95 masks used in three local hospitals: Dana-Farber Cancer Institute, Brigham and Women’s Hospital, and Boston Children’s Hospital. N95 masks of the same model available in different sizes (for example, the 3M 1860 and the 3M 1860S, representing regular and small sizes) were considered to be interchangeable for testing purposes. The total sample size was necessarily limited by existing mask shortages and the importance of prioritizing the needs of healthcare workers; given the uniformity of the results reported below, the sample size was judged to be adequate.

Additionally, an assortment of other PPE and hospital equipment was selected for sterilization. This included the following PAPR components: Sentinel XL CBRN hood with hose, Sentinel head cover hoods, Sentinel PAPR breathing tubes for use with Sentinel XL HP PAPR (ILC Dover, Frederica, DE), and Bullard RT Series PAPR hood (Bullard, Lexington, KY). Other equipment included two models of face shields, one locally fabricated^24^ and the other a Fisherbrand Disposable Face Shield (Fisher Scientific, Waltham MA), a DuPont Tyvek 400 coverall (Wilmington, DE), an iPad (Apple, Cupertino CA), and an iPad case. The iPad was included for testing since BWH made them available to COVID-19 patients as a means of communicating with family members and there was an ongoing shortage of sterilizing wipes certified for use in this setting.

A first set of 30 N95 masks representing five different models was processed in the SteraMist system for zero to ten cycles, and then analyzed for single-pass filtration efficiency using ambient particulate matter at MIT. A second set of 44 N95 masks was processed using the SteraMist system and sent to ICS Laboratories for testing to an abbreviated (instantaneous only) or full loading NIOSH N-95 filtration efficiency protocol. Nine masks underwent a quantitative fit test at DFCI following sterilization.

### Sterilization in a SteraMist environment chamber

Sterilization of N95 masks and other PPE was accomplished using a SteraMist-equipped room (dimensions 5.64 m × 4.57 m × 3.05 m) at the DFCI animal research facility. Three SteraMist environmental units (room version TPO-302-PLC-V1.4) are mounted on the ceiling of the room and can be controlled via a single panel, accessible from the outside. iHP mist was delivered through three nozzles at a total of 90 mL/min for 15 min, yielding a delivered concentration of 17.7 mL/m^3^.

N95 masks were placed with their interior surfaces facing up on standard stainless-steel shelves (open grid, InterMetro style). Most of the other PPE were also laid out on these shelves with the exception of two PAPR hoods, one PAPR hose, and a Tyvek coverall, which were hung in various configurations (Supplementary Fig. S1). PPE was spaced 6 cm to 20 cm apart on each shelf; this was designed to test sterilization performance at multiple points in the chamber. Tighter but non-overlapping spacing would likely be necessary for processing equipment in higher volumes. However, as long as the chamber mist dwell time and iHP concentration are maintained at a constant level, tighter spacing should not affect the method’s ability to effectively disinfect masks.

Two PAPR hoods, one PAPR hose, and one face shield were pre-treated with a SteraMist handheld spraying device in advance of processing in the SteraMist-equipped chamber. Pre-treatment was intended to ensure delivery of sterilant to items with semi-enclosed surfaces (such as the inside of a PAPR hose). Staff who treated these items wore appropriate PPE, including goggles, an N95 respirator, and gloves, and used a handheld device to spray the equipment from a distance of approximately 0.5–1 m for a few seconds per surface. Per manufacturer protocol, the 100-min sterilization cycle in the environmental chamber included: program initiation, during which the inner and outer doors are locked to seal the room and a bubble damper closes over the exhaust to prevent air exchange; an initial 15-min fill phase during which the mist was released; a 20-min dwell phase to allow the mist to penetrate the room; a 65-min scrub phase during which the exhaust was re-opened to aerate the space at a rate of 43 air changes per hour; and program conclusion during which the room unseals. Following program conclusion, staff test for off-gassing using a PortaSens II Portable Gas Leak Detector Model C16 (Analytical Technology, Inc, Collegeville, PA) to ensure readings of H_2_O_2_ < 1 ppm at the entrance and center of the room. Masks samples from the front, middle, and back of the room were also tested for off-gassing before removal. This was accomplished by briefly placing each sample in a 1 mil thick polypropylene plastic bag (dimensions: 20.3 cm × 10.2 cm × 45.7 cm) and monitoring the air inside the bag for H_2_O_2_ using a PortaSens II Detector. The room is tested and calibrated quarterly for function by using enzymatic and biological indicators placed around the room (see below) and then ensuring homogeneous sterilization throughout the space.

### Evaluating sterility using biological indicators

The efficacy of sterilization was evaluated using Apex Biological Indicators (BIs: Mesa Labs; Boseman, MT); bacterial spores in these indicators are more resistant to killing than most viruses and therefore provide a conservative and simple estimate of sterilization efficacy. In particular, the *Geobacillus stearothermophilus* spores used in this study are known to be difficult to kill using hydrogen peroxide^6,25,26^. Each Apex biological indicator ribbon carries a minimum of 1.0 × 10^6^ *G. stearothermophilus* spores. The BIs were positioned in the environmental chamber prior to the first sterilization cycle. For N95 masks, BIs were placed under or adjacent to the masks. For the PAPR components and other equipment, BIs were placed on surfaces that were judged to be least accessible to the sterilant (for example, inside the PAPR tubing) (Supplementary Table S1). The BIs were extracted using sterile forceps after 1 treatment cycle, placed in Releasat growth medium (Mesa Labs), incubated at 55–60 °C, and monitored for bacterial growth over a period of 10 days using a colorimetric assay^27^. Previous work with *G. stearothermophilus* spores suggests that a conservative benchmark for complete sterilization represents a 6-log_10_ kill; that is, a ratio in the number of surviving to initial viable spores in a BI of 10^–6^ (Ref.^28^). This corresponds to no observable bacterial growth and thus no color change after 5 days in Releasat medium.

Use of standard bacterial indicators were chosen over tests with SARS-Cov-2 virus for three reasons. First, spores in bacterial indicators (BIs) are known to be substantially more resilient to hydroxyl radicals than enveloped viruses^6^ and it is well established that H_2_O_2_ is an effective a sterilant for virus similar to coronaviruses^29^. Second, the primary question being addressed with bacterial indicators in the current work is whether hydrogen peroxide vapor is sufficiently dispersed in the treatment chamber to reach all PPE regardless of variation in placement. Third, studies with live virus would not generally be accessible to routine users of iHP methods. Instead, BIs provide a conservative and well-established measure of sterilization efficiency.

### Evaluating filtration efficiency

Single-pass filtration efficiency testing was performed at MIT on five control N95 masks and 25 masks sterilized in the SteraMist-equipped chamber. Filtration efficiency results obtained from this apparatus were compared to those obtained to NIOSH specifications at a commercial pre-certification laboratory (ICS Laboratories). Results with US-manufactured N95 masks (n = 10) exhibited good concordance between instantaneous filtration efficiency values measured using the MIT and ICS Lab tests, with a Pearson correlation coefficient of 0.89 (p = 0.0006). Additional information on the testing apparatus is available at cleanmask.org and in the literature^30^.

For filtration testing at MIT, 8 cm × 8 cm sample of each N95 mask was inserted into a specialized air duct, with a cross-sectional area of 50.3 cm^2^ (Supplementary Fig. S2), and ambient particulate matter was driven through the duct, and thus through the mask fabric, using a pressure differential of ~ 175 pascals at 0.4 m/s face velocity. The concentration of 0.3, 0.5, and 1 μm diameter particles prior to and after passage through the mask fabric was determined using an Aerotrak 9306 optical particle counter (TSI Inc.; Shoreview, MN) (Table 2). Filtration efficiency testing was performed a second time on a subset of decontaminated masks stored for 10 days after treatment to test for time-dependent degradation in N95 mask performance following sterilization. Although readily available, the testing performed at MIT is not equivalent to NIOSH-approved testing for N95 masks and thus, these results should be interpreted as a relative, not absolute, measurements of filtration efficiency.

**Table 2:** Results obtained at a university laboratory on single-pass filtration efficiency for ambient particle matter. Each row represents a single N95 mask. Filtration efficiency values are an average of four upstream and downstream measurements. Standard deviations are calculated from repeat measurements made on a single mask.

Model	Cycles	Filtration Efficiency (SD, %)		
		0.3 μm	0.5 μm	1.0 μm
3M 1860	0	97.66% (0.18)	99.05% (0.13)	99.68% (0.03)
	0	97.53% (0.18)	99.11% (0.23)	100.00% (0.00)
	1	99.20% (0.08)	99.70% (0.08)	99.90% (0.20)
	2	98.98% (0.10)	99.80% (0.06)	100.00% (0.00)
	2	99.42% (0.05)	99.89% (0.09)	99.91% (0.18)
	3	99.36% (0.12)	99.92% (0.06)	100.00% (0.00)
	4	98.55% (0.07)	99.59% (0.13)	99.88% (0.24)
	5	98.76% (0.03)	99.52% (0.16)	100.00% (0.00)
	10	98.45% (0.15)	99.39% (0.09)	100.00% (0.00)
KC/Halyard 46767 (duckbill)	0	99.91% (0.02)	99.95% (0.05)	100.00% (0.00)
	1	99.83% (0.07)	99.86% (0.17)	100.00% (0.00)
	2	99.91% (0.02)	99.98% (0.02)	100.00% (0.00)
	3	99.90% (0.04)	99.98% (0.04)	100.00% (0.00)
	4	99.69% (0.06)	99.80% (0.12)	99.89% (0.24)
	5	99.89% (0.03)	99.95% (0.07)	100.00% (0.00)
	10	99.86% (0.07)	99.97% (0.06)	100.00% (0.00)
Gerson 2130	1	96.06% (0.20)	98.90% (0.10)	99.68% (0.43)
	2	96.46% (0.19)	99.08% (0.11)	99.84% (0.19)
	3	95.17% (0.37)	98.80% (0.26)	99.65% (0.30)
3M 8210	0	98.09% (0.22)	99.42% (0.24)	99.82% (0.21)
	1	99.86% (0.04)	99.99% (0.02)	100.00% (0.00)
	2	99.52% (0.03)	99.93% (0.04)	100.00% (0.00)
	3	99.28% (0.06)	99.88% (0.04)	100.00% (0.00)
	4	98.90% (0.11)	99.40% (0.10)	100.00% (0.00)
	10	99.16% (0.15)	99.77% (0.13)	100.00% (0.00)
3M 9210/37021	0	99.75% (0.11)	99.92% (0.11)	100.00% (0.00)
	1	99.77% (0.16)	99.83% (0.19)	99.71% (0.37)
	2	99.70% (0.07)	99.92% (0.07)	100.00% (0.00)
	3	99.39% (0.18)	99.86% (0.04)	100.00% (0.00)
	4	98.68% (0.98)	99.01% (0.92)	99.05% (1.19)

A second sample of sterilized N95 masks was tested at ICS labs to NIOSH standards with 34 masks undergoing an instantaneous filter efficiency test and 10 masks undergoing a full loading filter efficiency test. Testing was performed using a TSI Automated Filter Tester Model 8130A (TSI Inc., Shoreview, Minnesota). Per NIOSH testing Procedure No. TEB-APR-STP-0059 (rev. 3.2)^31^, all masks were challenged with a sodium chloride aerosol neutralized to a Boltzmann equilibrium state at 25 ± 5 °C and a relative humidity of 30 ± 10%. Particle size and distribution was verified to correspond to a median diameter of 0.075 ± 0.020 µm, with a geometric standard deviation ≤ 1.86. N95 masks were conditioned at 85 ± 5% relative humidity and 38 ± 2 °C for 25 h prior to filter efficiency testing. For instantaneous filter efficiency testing, each mask was then assembled into a fixture and subjected to instantaneous aerosol loading. The loading was performed by depositing sodium chloride aerosol at an airflow rate of 85 L per minute (LPM) for one minute. For full loading filter efficiency, each mask was assembled into a fixture and subjected to full aerosol loading. The loading was performed by depositing 200 mg of sodium chloride aerosol at an airflow rate of 85 LPM for 75 min. Flow rate was monitored every 5–10 min on average and adjusted to maintain a flow rate of 85 ± 2 LPM.

### Quantitative fit testing

Nine masks from three models underwent a quantitative fit test following 2, 5, and 10 sterilization cycles to confirm that sterilization did not interfere with the ability of masks to form an effective seal with the human face. Testing was performed with the full set of OSHA prescribed wearer exercises using a PortaCount Pro + 8038 fit tester (TSI Inc.; Shoreview, MN) set to the 100–200 fit factor range, per manufacturer recommendation.

### Ethics approval

Ethics approval was not necessary to conduct this study.

## Results

### Evaluating sterilization using biological indicators

All BIs placed under or adjacent to N95 masks that had been exposed to a single sterilization cycle in the SteraMist-equipped chamber exhibited at least a 9-log_10_ kill (representing no color change following seven days of incubation in Releasat medium). BIs placed within PAPR hoods also achieved 9-log_10_ kill as did a BI placed in a PAPR hose that was pre-treated using a SteraMist handheld spraying device (Supplementary Table S1). BIs placed on the iPad, iPad case, and PanFab face shield designs^24^ all passed the sterilization threshold, and the iPad was observed to be fully functional after one cycle of iHP treatment. In contrast, two BIs placed inside either end of a PAPR hose that was not subjected to pre-treatment were not sterilized, as determined by rapid bacterial growth following transfer to Releasat medium. This was also true of a BI embedded in the thick foam at the top of a Fisherbrand Disposable Face Shield. We tested the effect of pretreating the same face shield with a hand-held SteraMist device (after inserting a new BI) and observed a 4-log_10_ kill, which also fails the 6-log_10_ threshold conventionally used to score successful sterilization.

From these data we conclude that a single iHP cycle is efficacious at sterilizing N95 masks and other equipment having readily accessible surfaces when the equipment is placed throughout a SteraMist-equipped decontamination chamber. Moreover, the process is not obviously damaging to delicate equipment such as an iPad (n = 1). Penetration into semi-enclosed spaces such as PAPR hoses appeared to be less efficient, but such equipment could be sterilized by pre-treatment with a handheld iHP-delivery device followed by a cycle of iHP treatment in a chamber. This suggests that forced ventilation of hoses, with a small fan for example, would enable hands-free sterilization of hoses but we were unable to test this possibility. We found that iHP vapor did not penetrate 2.5 cm foam on a disposable face shield at sufficient concentrations to sterilize biological indicators, even following pre-treatment with a handheld device. These data suggest that normally disposable face shields should not be reused. In contrast, a custom-fabricated face shield^24^ introduced under an FDA EUA and consisting of 3D printed parts and closed cell Ethylene-Vinyl Acetate (EVA) foam appeared to be sterilized effectively. Further research is required to understand the factors (e.g. open vs. closed cell construction) that determine whether foams and similarly compliant materials can be sterilized with iHP vapor.

### Evaluating filtration efficiency

Performance data was collected at MIT on five models of N95 masks from three manufacturers (a total of 30 units) using an ambient particulate matter filtration efficiency test. Relative to control N95 masks, we observed no reduction in filtration efficiency for 0.3, 0.5, and 1 µm particles by N95 masks subjected to up to ten sterilization cycles (Table 2). Data on pressure, temperature, air face velocity, and relative humidity during testing are found in Supplementary Table S2.

**Figure 1:**
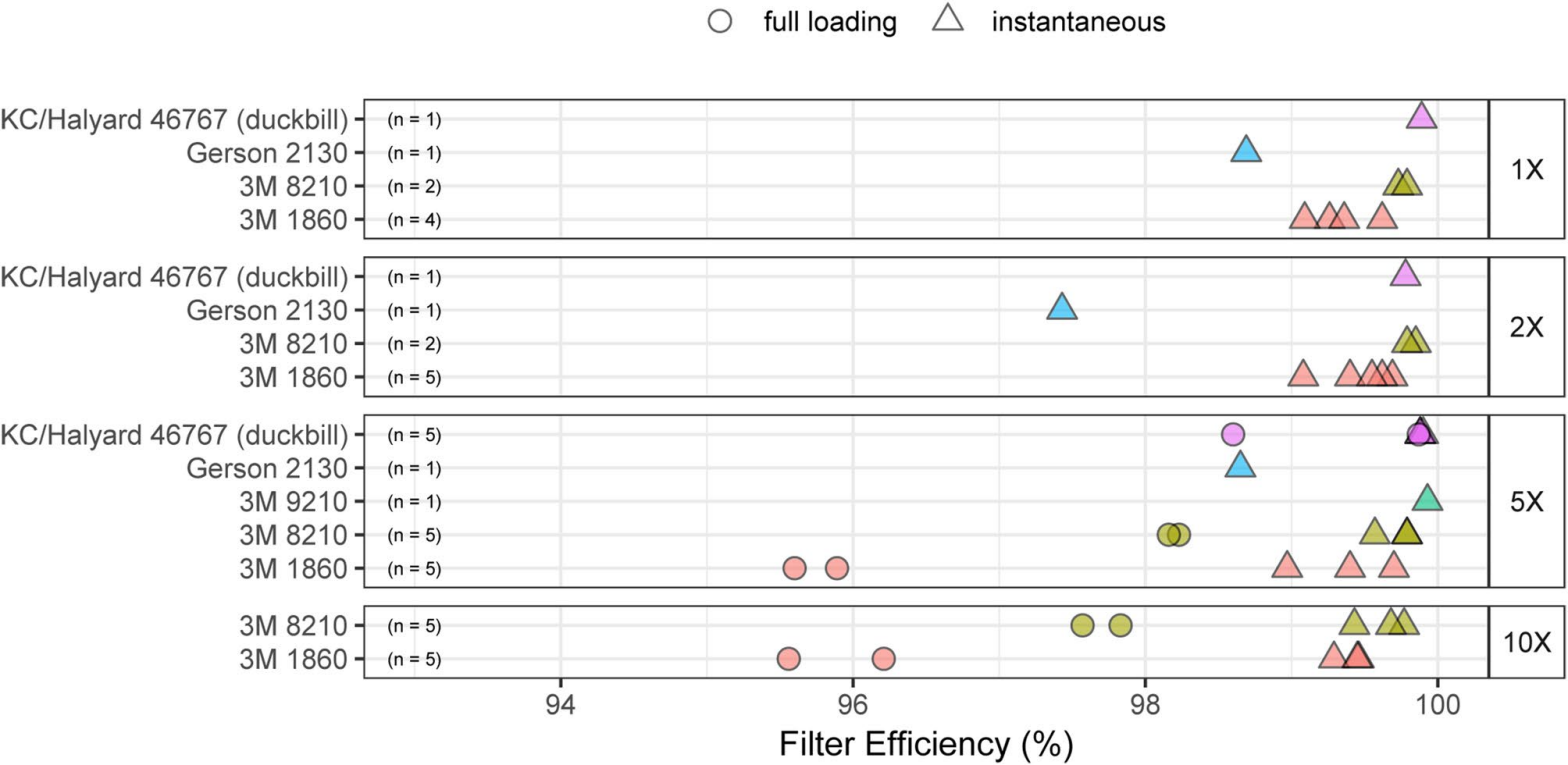
Instantaneous and fully-loaded ambient particulate matter filtration data for N95 masks over one, two, five, and 10 SteraMist sterilization cycles (1 ×, 2 ×, 5 ×, and 10 × respectively). “KC” corresponds to Kimberly-Clark. Figure marker color corresponds to mask model type (listed on y-axis). Results were obtained from ICS Laboratories according to NIOSH standard Procedure No. TEB-APR-STP-0059. All masks passed ICS standards, including filtration efficiency of ≥ 95%.

In addition, five N95 mask models from three manufacturers (44 units total) were evaluated using testing protocols derived from NIOSH published standard testing procedures (STPs) maintained by ICS Laboratories. These data showed that all 34 iHP-sterilized N95 masks retained instantaneous filtration efficiencies of ≥ 95%, including masks subjected to ten sterilization cycles, the maximum number of cycles tested (Fig. 1, Tables 3, 4, and Supplementary Table S3). Gerson 2130 N95 masks were the least effective at filtering 75 nm NaCl particles, but even these units passed the instantaneous test threshold out to five sterilization cycles (the maximum number tested for this model). Per NIOSH standards, inhalation resistance should not exceed 35 mm of H_2_O and exhalation resistance should not exceed 25 mm of H_2_O^32^. No mask exceeded these thresholds, and in no case did users perceive increased resistance to airflow during normal breathing.

**Table 3:** Results from ICS Laboratories on instantaneous filtration efficiency according to NIOSH standard Procedure No. TEB-APR-STP-0059 (the flow rate was 85–86 LPM). Each row represents data from one to five N95 masks of a particular model and data are reported as the average for all tests that were performed. In contrast to the data in Table 2, each mask was measured only once and standard deviations are therefore reported only when the number of masks was greater than one.

Model	Cycles	Number of masks	Resistance (mm of H_2_O)	Penetration (%)	Filter efficiency (SD, %)
3M 1860	1	4	10.35	0.67	99.33 (0.22)
	2	5	9.78	0.53	99.47 (0.24)
	5	3	9.10	0.64	99.36 (0.37)
	10	3	9.17	0.60	99.40 (0.10)
KC/Halyard 46767 (duckbill)	1	1	15.20	0.11	99.89
	2	1	14.10	0.22	99.78
	5	3	14.33	0.11	99.89 (0.01)
Gerson 2130	1	1	9.30	1.31	98.69
	2	1	7.90	2.57	97.43
	5	1	9.80	1.35	98.65
3M 8210	1	2	8.85	0.24	99.76 (0.04)
	2	2	8.95	0.18	99.82 (0.04)
	5	3	9.40	0.28	99.72 (0.13)
	10	3	7.77	0.37	99.63 (0.18)
3M 9210/37021	5	1	10.40	0.07	99.93

**Table 4:** Results from ICS Laboratories on full loading filtration efficiency according to NIOSH standard Procedure No. TEB-APR-STP-0059. Each row represents data from a single measurement on a single N95 mask.

Model	Cycles	Resistance (mm of H_2_O)	Initial penetration (%)	Maximum penetration (%)	Filter efficiency (%)
3M 1860	5	9.6	1.22	4.40	95.60
	5	9.8	0.94	4.11	95.89
	10	8.3	0.67	4.44	95.56
	10	8.9	0.62	3.79	96.21
KC/Halyard 46767 (duckbill)	5	15.5	1.40	1.40	98.60
	5	14.8	0.13	0.13	99.87
3M 8210	5	9.8	0.29	1.77	98.23
	5	9.9	0.49	1.84	98.16
	10	8.5	0.21	2.43	97.57
	10	10	0.23	2.17	97.83

Fully loaded filtration efficiency was also evaluated by ICS Laboratories to NIOSH standards. The purpose of this test is to mimic the effect of an accumulation of charged particles in a mask, a phenomenon related to time of use. Mask loading is known to lower filtration efficiency, potentially by reducing the electrostatic charge of fabric in the filtering layer^33^. Again, we observed that the 10 sterilized masks subjected to this test passed the NIOSH threshold for N95 pre-certification.

To test for time-dependent degradation of performance, 26 N95 masks were stored for ten days after initial filtration testing and then re-tested using the MIT apparatus (Supplementary Table S4); the time between iHP sterilization and retesting varied between 10–15 days depending on cycle number. We observed no difference in filtration performance between measurements taken immediately after sterilization, and those taken 10 days after, as determined by a repeated measures ANOVA (p = 0.45). From these data we conclude that the filtration efficiency of multiple models of N95 masks is not substantially affected by one to ten cycles of iHP sterilization in terms of filtration efficiency or inhalation resistance, and that all masks tested meet existing NIOSH pre-certification standards.

### PortaCount quantitative fit data

All nine masks that were subjected to quantitative fit testing (KC/Halyard 46767, 3M 1860, 3M 8210) using PortaCount equipment achieved a passing value of > 200 fit factor following 2, 5, and 10 sterilization cycles. This corresponds to a filtration efficiency of 99% or higher (data not shown) according to manufacturer guidelines. Thus, iHP sterilization does not appear to impair the ability of N95 masks to form an effective seal against a user’s face.

## Discussion

Hydrogen peroxide has a long history of successful use in the field of medical device sterilization, and our results support the use of iHP as a PPE sterilant when delivered using a SteraMist-equipped environment chamber, in some cases complemented by pre-treatment with a handheld iHP delivery device. Thus, iHP sterilization can likely be used to extend the usability of PPE such as N95 masks that are usually disposed of after a single use. The DFCI SteraMist environment chamber used in this study has a volume of ~ 80 m^3^ and could comfortably fit ~ 2400 N95 masks per cycle without the masks touching each other, or a lesser number of PAPR hoods and other PPE. At this rate, assuming idealized staffing and logistics, roughly 4800–7200 masks could be sterilized for use per day given a typical 100 min sterilization cycle. These numbers could be increased with the addition of an overnight workforce.

In keeping with standard practice, sterility was judged in this study using biological indicators containing bacterial spores and was not based on killing of pathogens such as SARS-CoV-2 encountered in a clinical setting. However, the *G. stearothermophilus* spores in the BIs we used are known to be resistant to killing by hydrogen peroxide, and spores are substantially more resistant to sterilization than enveloped viruses such as SARS-CoV-2^34^. Recent work has also demonstrated that VHP can kill SARS-CoV-2^11,19,35^. Thus, we do not believe that our use of BIs rather than direct measurement of viral viability represents a significant limitation in the interpretation of the data.

Two independent lines of evidence, one generated at a university laboratory and one at a commercial laboratory accredited to perform N95 mask certification to NIOSH/ISO/IEC standards, show that N95 masks decontaminated with iHP using SteraMist technology retain their performance with respect to filtration and inhalation resistance for at least ten cycles, the maximum number tested. No deterioration was detected in masks tested 10 days post treatment. Quantitative fit testing of N95 masks sterilized up to ten cycles confirmed that they still form an airtight seal as required. Thus, sterilized N95 masks remain fully functional.

### Limitations of this study

The tests described in this study were conducted using unused N95 masks. We do not yet have data on N95 masks used in an actual health care facility responding to a pandemic. Such studies are important for assessing the real-world implication of mask reuse. Previous work has found that masks subjected to multiple donnings and doffings fail fit testing^36^ although potential solutions to this problem have been proposed^37^. Additional questions that must be addressed by real-world testing include inhalation resistance for an N95 mask that has been loaded with internal and external contaminants, the comfort level of health care workers in using an N95 mask that is sterilized but previously used by another individual, and the rate of wastage arising from breakage of elastic bands, and contamination with makeup or topical face products. These types of real-world use data are not available for any iHP/VHP-based sterilization method, even for technologies that have been promoted commercially, in part because testing masks potentially contaminated with SARS-CoV-2 is not feasible on standard equipment. Nonetheless, the data reported in this study were judged by our clinical teams to be sufficient to implement N95 mask sterilization and reuse at DFCI.

Future work should address the question of whether decontaminated N95 masks must be returned to the original users (as specified in the Sterrad and Sterris EUAs for N95 mask decontamination) or can be returned to a common pool (as specified in the Batelle EUA); the latter is substantially easier to implement from a logistical perspective. Additional work is also required to determine whether mechanical ventilation would make it possible to sterilize PAPR hoses and similar devices without the need for manual pre-treatment. Additionally, while there is good support for the use of spore-based BIs in measuring the efficacy of sterilization, direct tests on SARS-CoV-2 itself may be warranted, particularly in the case of items such as hoses and other PPE that have a complex shape. Finally, while we determined that masks did not release detectable H_2_O_2_ following sterilization, we did not assess the effect of time after sterilization on off-gassing: we simply used the manufacturer’s recommended venting protocol.

## Conclusions

Our data support the use of the SteraMist iHP technology as a sterilization method for reuse of N95 masks, including many of the most commonly used models, as well as some other types of PPE—in some cases following pre-treatment with an iHP handheld delivery device. In interpreting these data, it is important to note that not all iHP/VHP methods are the same. While Bioquell is approved under an FDA EUA for 20 cycles, N95 masks sterilized using an alternative HPGP method commercialized by Sterrad fail at five cycles (the Sterrad EUA was approved for 2 sterilization cycles and requires that a mask be returned to a single user)^11,38^. Moreover, our data show that semi-enclosed items of PPE, such as PAPR hoses, cannot be sterilized without pre-treatment, and that face shields with thick foam may not be sterile even after exposure to iHP using a handheld device followed by an environmental chamber. Thus, it is imperative that institutions seeking to deploy iHP/VHP technology review primary data prior to local deployment. We also suggest that BIs routinely be deployed to ensure equipment performance.

The issues attending reuse of N95 masks have been recognized for over two decades based on multiple instances of human transmission of novel respiratory diseases. As the global response to COVID-19 evolves, we hope that the study of sterilization technologies such as iHP/VHP will continue and involve peer-review of independently acquired data so that we are in a better position for coming waves of the current pandemic and possible pandemics in the future.

## Supplementary Information

Supplementary Information.

## Data Availability

All data relevant to the study are included in the article or uploaded as supplementary information.
