# Supplementary material for: Analysis of SteraMist ionized hydrogen peroxide technology in the sterilization of N95 respirators and other PPE
Source: Sci Rep. 2021 Jan 21;11:2051. doi: 10.1038/s41598-021-81365-7 (PMC7819989; doi:10.1038/s41598-021-81365-7)
Supplement: Supplementary file 1 — Supplementary Information. [file 41598_2021_81365_MOESM1_ESM.pdf]

Supplementary Materials

Analysis of SteraMist ionized hydrogen peroxide technology in the sterilization of N95 respirators and other PPE

Avilash K. Cramer*, Deborah Plana*, Helen Yang*, Mary M. Carmack, Enze Tian, Michael S. Sinha, David Krikorian, David Turner, Jinhan Mo, Ju Li, Rajiv Gupta, Heather Manning, Florence T. Bourgeois, Sherry H. Yu, Peter K. Sorger, and Nicole R. LeBoeuf**.**

[*]These authors contributed equally to this work
[**]These authors contributed equally to this work

Supplementary Figure S1: Diagram illustrating the testing set-up for SteraMist sterilization, with accompanying representative images. The chamber has an overall volume of ~80 m^3.

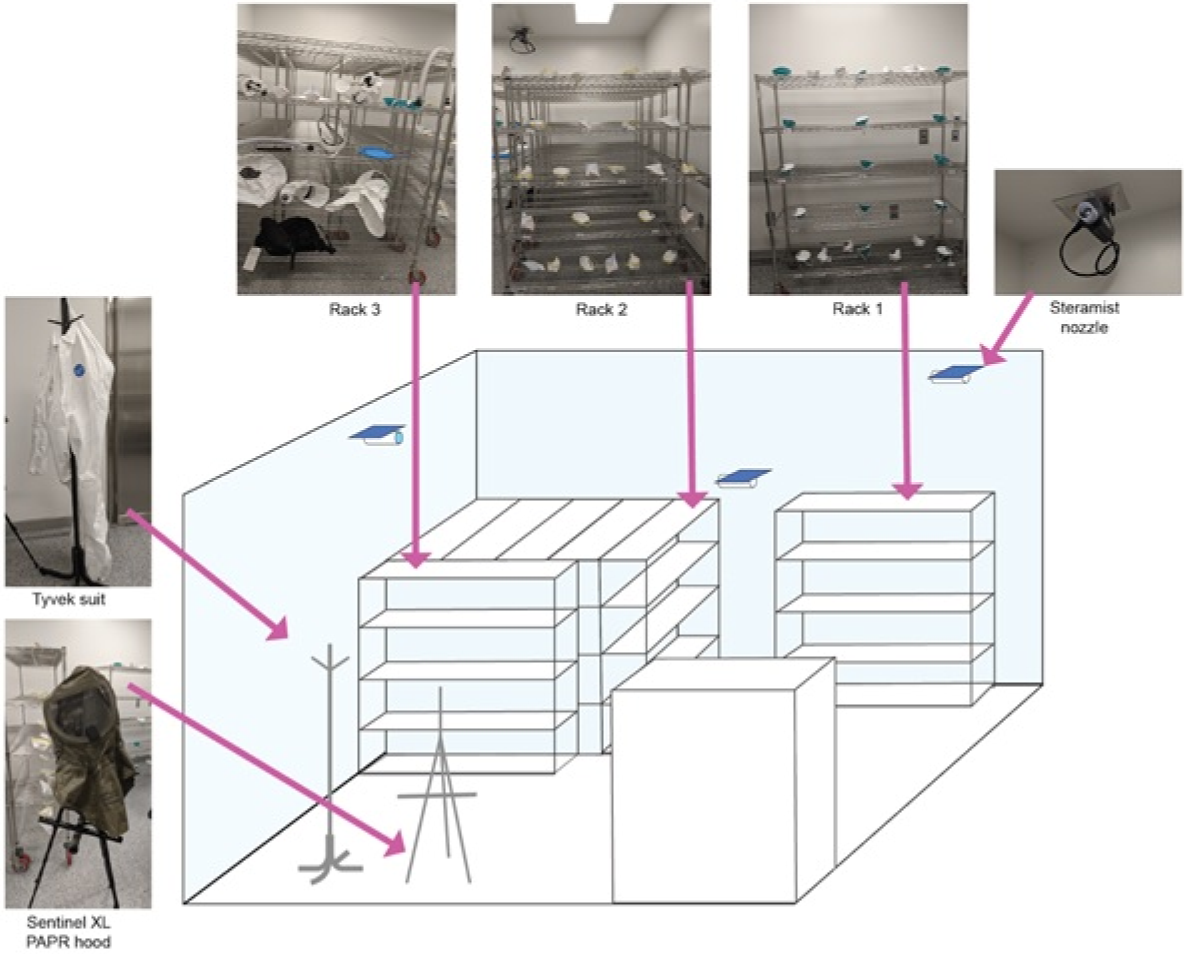

Supplementary Figure S2: Image of ambient particulate matter air ducts used in MIT testing of N95 masks. Note that this is a destructive test in which the N95 mask is cut prior to introduction into the testing chamber.

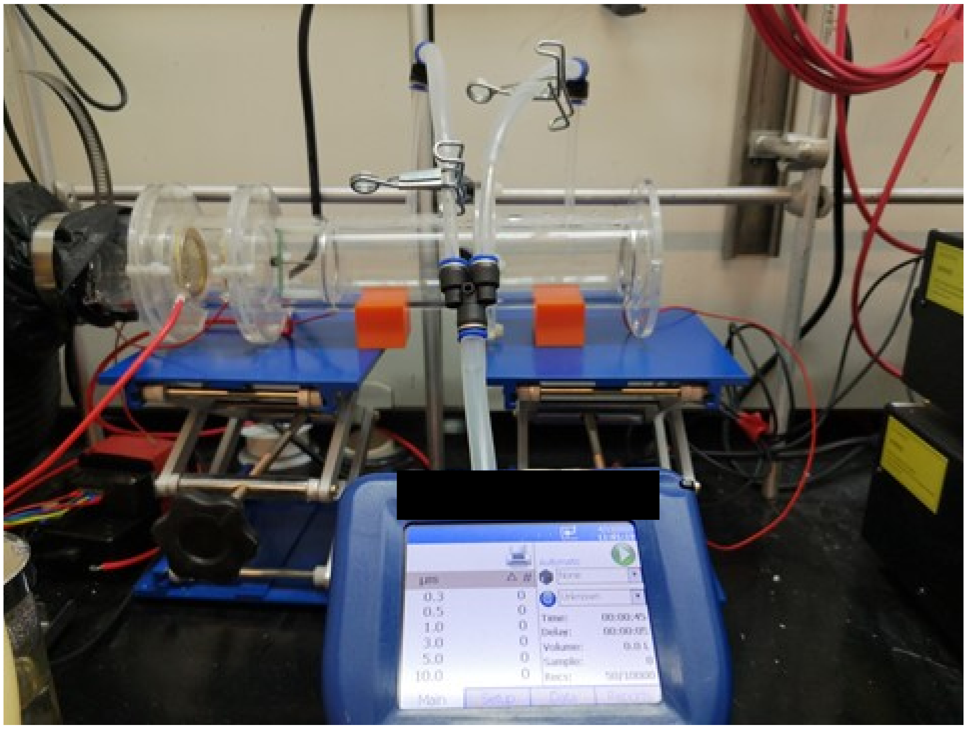

Supplementary Table S1: Results of biological indicator (BI) monitoring after one-cycle of sterilization in a SteraMist-equipped environment chamber.

**Representative PPE items and BI location**	**Was surface pre-treated?**	**7-day 9-log_{10} kill result**	**BI test location**
Sentinel® XL CBRN PAPR hood and hose	Yes	Passed	Inside top surface; under hose flap
Sentinel® PAPR hood head cover	No	Passed	Inside top surface
Sentinel® PAPR hood head cover	Yes	Passed	Chin strap; inside top surface
Sentinel® PAPR breathing tube for use with Sentinel XL HP PAPR	No	Failed	On front end of hose; on back end of hose
Sentinel® PAPR breathing tube for use with Sentinel XL HP PAPR	Yes	Passed	Inside tube
Bullard RT Series PAPR hood	No	Passed	Inside top surface; outside top surface
DuPont Tyvek® 400 coverall	Yes	Passed	Outside chest; outside surface of axilla; inside surface of axilla
Fisherbrand face shield	No	Failed	Embedded in foam
Fisherbrand face shield	Yes	Failed (note: passed at 4-log_{10} kill)	Embedded in foam
3D printed face shield (BWH/PanFab)	No	Passed	On Shield
iPad and case	No	Passed	On device; on case
N95 masks	No	Passed	Located on racks 1 and 2 on top middle, and bottom shelves

Supplementary Table S2: Results of ambient particulate matter filtration efficiency evaluation performed at MIT, recorded within 24 hours of treatment. Values are an average of four upstream and downstream measurements, with standard deviation (SD) shown.

Model	Cycles	Filtration Efficiency (SD)			ΔP (pa)	V_{air} (m/s)	T (°C)	Relative Humidity (%)
		0.3μm	0.5μm	1.0μm				
**3M 1860**	0 (control)	97.66% (0.18)	99.05% (0.13)	99.68% (0.03)	186.1	0.3	24.1	22.5
	0 (control)	97.53% (0.18)	99.11% (0.23)	100.00% (0.00)	181.1	0.4	23.2	26.2
	1	99.20% (0.08)	99.70% (0.08)	99.90% (0.20)	191.5	0.024-0.1	24.3	22.0
	2	98.98% (0.10)	99.80% (0.06)	100.00% (0.00)	194.0	0.024-0.1	24.8	21.4
	2	99.42% (0.05)	99.89% (0.09)	99.91% (0.18)	192.0	0.024-0.1	24.8	21.4
	3	99.36% (0.12)	99.92% (0.06)	100.00% (0.00)	193.0	0.024-0.1	25.2	20.3
	4	98.55% (0.07)	99.59% (0.13)	99.88% (0.24)	192.6	0.3	25.1	20.2
	5	98.76% (0.03)	99.52% (0.16)	100.00% (0.00)	186.6	0.024-0.1	23.3	31.1
	10	98.45% (0.15)	99.39% (0.09)	100.00% (0.00)	180.2	0.4	23.8	25.5
**KC/Halyard 46767 (duckbill)**	0 (control)	99.91% (0.02)	99.95% (0.05)	100.00% (0.00)	190.1	0.024-0.1	24.3	22.7
	1	99.83% (0.07)	99.86% (0.17)	100.00% (0.00)	192.6	0.024-0.1	24.8	21.7
	2	99.91% (0.02)	99.98% (0.02)	100.00% (0.00)	193.2	0.024-0.1	24.9	22.1
	3	99.90% (0.04)	99.98% (0.04)	100.00% (0.00)	194.3	0.024-0.1	24.7	21.0
	4	99.69% (0.06)	99.80% (0.12)	99.89% (0.24)	194.5	0.024-0.1	24.8	20.5
	5	99.89% (0.03)	99.95% (0.07)	100.00% (0.00)	186.6	0.024-0.1	22.7	32.0
	10	99.86% (0.07)	99.97% (0.06)	100.00% (0.00)	186.4	0.024-0.1	23.8	25.4
**Gerson 2130**	1	96.06% (0.20)	98.90% (0.10)	99.68% (0.43)	194.0	0.024-0.1	24.8	21.4
	2	96.46% (0.19)	99.08% (0.11)	99.84% (0.19)	193.3	0.024-0.1	24.7	21.7
	3	95.17% (0.37)	98.80% (0.26)	99.65% (0.30)	192.4	0.024-0.1	25.0	20.7
**3M 8210**	0 (control)	98.09% (0.22)	99.42% (0.24)	99.82% (0.21)	187.8	0.3	24.6	22.1
	1	99.86% (0.04)	99.99% (0.02)	100.00% (0.00)	192.0	0.024-0.1	24.8	21.7
	2	99.52% (0.03)	99.93% (0.04)	100.00% (0.00)	191.2	0.024-0.1	24.9	21.3
	3	99.28% (0.06)	99.88% (0.04)	100.00% (0.00)	191.4	0.3	25.0	20.3
	4	98.90% (0.11)	99.40% (0.10)	100.00% (0.00)	193.0	0.3	25.2	20.0
	10	99.16% (0.15)	99.77% (0.13)	100.00% (0.00)	179.4	0.4	23.8	25.2
**3M 9210/37021**	0 (control)	99.75% (0.11)	99.92% (0.11)	100.00% (0.00)	190.6	0.024-0.1	24.6	21.9
	1	99.77% (0.16)	99.83% (0.19)	99.71% (0.37)	193.8	0.024-0.1	24.8	21.5
	2	99.70% (0.07)	99.92% (0.07)	100.00% (0.00)	192.4	0.2	24.9	20.9
	3	99.39% (0.18)	99.86% (0.04)	100.00% (0.00)	192.0	0.024-0.1	25.0	20.6
	4	98.68% (0.98)	99.01% (0.92)	99.05% (1.19)	192.8	0.024-0.1	24.9	20.6

Supplementary Table S3: Results of instantaneous filtration efficiency evaluation performed at ICS Laboratories (Brunswick, OH).

Model	Cycle	Flow Rate (LPM)	Resistance (mm of H2O)	Penetration (%)	Filter Efficiency (%)
**3M 1860**	1	86	10.4	0.74	99.26
	1	86	9.5	0.38	99.62
	1	86	10.1	0.91	99.09
	1	86	11.4	0.64	99.36
	2	86	10.9	0.92	99.08
	2	86	9.3	0.60	99.40
	2	86	9.9	0.31	99.69
	2	86	9.6	0.38	99.62
	2	86	9.2	0.45	99.55
	5	86	9.1	1.03	98.97
	5	86	8.8	0.60	99.40
	5	86	9.4	0.30	99.70
	10	86	10.7	0.54	99.46
	10	86	8.4	0.71	99.29
	10	86	8.4	0.55	99.45
**KC/Halyard 46767 (duckbill)**	1	86	15.2	0.11	99.89
	2	86	14.1	0.22	99.78
	5	86	14.5	0.12	99.88
	5	86	13.5	0.10	99.90
	5	86	15.0	0.12	99.88
**Gerson 2130**	1	86	9.3	1.31	98.69
	2	86	7.9	2.57	97.43
	5	85	9.8	1.35	98.65
**3M 8210**	1	86	8.9	0.27	99.73
	1	86	8.8	0.21	99.79
	2	86	8.6	0.15	99.85
	2	86	9.3	0.21	99.79
	5	85	9.3	0.21	99.79
	5	85	9.1	0.43	99.57
	5	85	9.8	0.21	99.79
	10	86	7.9	0.23	99.77
	10	86	7.4	0.32	99.68
	10	86	8.0	0.57	99.43
**3M 9210/37021**	5	85	10.4	0.07	99.93

Supplementary Table S4: Results of ambient particulate matter filtration efficiency evaluation re-performed at MIT 10 days after sterilization treatment. Values are an average of four upstream and downstream measurements.

Model	Cycles	0.3µm (SD)	0.5µm (SD)	1.0µm (SD)	ΔP (pa)	V_{air} (m/s)	T (°C)	Relative Humidity (%)
**3M 1860**	0 (control)	95.84% (0.18)	98.41% (0.52)	100.00% (0.00)	183.7	0.3	24.7	15.2
	1	99.31% (0.04)	99.76% (0.13)	100.00% (0.00)	183.2	0.024-0.1	25.2	14.4
	2	97.71% (0.08)	99.02% (0.30)	100.00% (0.00)	184.2	0.024-0.1	25.3	14.3
	2	99.39% (0.09)	99.68% (0.23)	100.00% (0.00)	184.8	0.024-0.1	25.4	14.4
	3	99.08% (0.01)	99.58% (0.42)	100.00% (0.00)	183.0	0.3	25.2	14.9
	4	97.91% (0.11)	99.17% (0.36)	100.00% (0.00)	182.6	0.3	25.3	14.7
	5	98.43% (0.03)	99.33% (0.30)	100.00% (0.00)	183.6	0.3	25.3	14.7
**KC/Halyard 46767 (duckbill)**	0 (control)	99.86% (0.02)	100.00% (0.00)	100.00% (0.00)	186.0	0.024-0.1	25.2	15.1
	1	99.87% (0.03)	99.97% (0.05)	100.00% (0.00)	186.5	0.024-0.1	25.3	13.9
	2	99.92% (0.02)	100.00% (0.00)	100.00% (0.00)	185.9	0.024-0.1	25.4	13.7
	3	99.91% (0.03)	99.98% (0.05)	100.00% (0.00)	184.8	0.024-0.1	24.8	15.6
	4	99.76% (0.01)	99.95% (0.09)	100.00% (0.00)	183.8	0.024-0.1	25.1	15.1
	5	99.82% (0.06)	100.00% (0.00)	100.00% (0.00)	184.4	0.024-0.1	25.3	14.8
**Gerson 2130**	1	95.62% (0.15)	98.24% (0.33)	100.00% (0.00)	186.2	0.024-0.1	25.3	14.1
	2	95.38% (0.25)	98.27% (0.19)	100.00% (0.00)	185.4	0.024-0.1	25.3	14.0
	3	93.81% (0.15)	96.99% (0.41)	100.00% (0.00)	184.2	0.024-0.1	25.3	14.8
**3M 8210**	0 (control)	98.69% (0.04)	99.45% (0.31)	100.00% (0.00)	181.1	0.4	25.3	14.7
	1	99.81% (0.03)	99.92% (0.11)	100.00% (0.00)	184.0	0.3	25.4	13.8
	2	99.40% (0.08)	99.76% (0.14)	100.00% (0.00)	184.8	0.024-0.1	25.4	13.8

	3	99.17% (0.02)	99.80% (0.18)	100.00% (0.00)	183.8	0.4	25.3	14.7
	4	99.13% (0.02)	99.71% (0.20)	100.00% (0.00)	183.6	0.3	25.3	14.6
**3M 9210/37021**	0 (control)	99.83% (0.01)	99.98% (0.05)	100.00% (0.00)	186.1	0.024-0.1	25.2	14.6
	1	99.92% (0.03)	99.95% (0.06)	100.00% (0.00)	186.0	0.024-0.1	25.3	13.9
	2	99.77% (0.03)	99.95% (0.05)	100.00% (0.00)	182.0	0.024-0.1	24.3	16.0
	3	99.19% (0.03)	99.64% (0.23)	100.00% (0.00)	184.4	0.024-0.1	25.3	14.6
	4	99.50% (0.05)	99.73% (0.15)	99.03% (1.67)	184.0	0.024-0.1	25.3	14.6
